# Design of a randomized controlled trial to evaluate effectiveness of methotrexate versus prednisone as first-line treatment for pulmonary sarcoidosis: the PREDMETH study

**DOI:** 10.1186/s12890-020-01290-9

**Published:** 2020-10-19

**Authors:** Vivienne Kahlmann, Montse Janssen Bonás, Catharina C. Moor, Coline H. M. van Moorsel, Mirjam Kool, Raisa Kraaijvanger, Jan C. Grutters, Mayka Overgaauw, Marcel Veltkamp, Marlies S. Wijsenbeek, Collaborating investigators, B. Koopman, J. J. M. Geelhoed, R. Janssen, R.E. Jonkers, H. Kramer, L. Moonen, R. L. M. Mostard, E. J. Nossent, M. J. Overbeek, R.H. N. A. J. van Rijswijk, M. Wagenaar, S. Walen, P. L. M. L. Wielders, D. W. Loth, B. A. H. A. Bogaarts, J. van der Maten

**Affiliations:** 1 Department of Respiratory Medicine, Centre of Excellence for Interstitial Lung Diseases and Sarcoidosis, Erasmus Medical Center, Rotterdam, the Netherlands; 2 Department of Pulmonology, ILD Center of Excellence, St. Antonius Hospital, Nieuwegein, the Netherlands; 3 Division of Heart and Lungs, University Medical Center Utrecht, Utrecht, The Netherlands; 4 Sarcoidosis patient association, Sarcoidose.nl, Alkmaar, the Netherlands

**Keywords:** Sarcoidosis, Prednisone, Methotrexate, Biomarkers, Quality of life, Home monitoring

## Abstract

**Background:**

Treatment of pulmonary sarcoidosis is recommended in case of significant symptoms, impaired or deteriorating lung function. Evidence-based treatment recommendations are limited and largely based on expert opinion. Prednisone is currently the first-choice therapy and leads to short-term improvement of lung function. Unfortunately, prednisone often has side-effects and may be associated with impaired quality of life. Methotrexate is presently considered second-line therapy, and appears to have fewer side-effects.

**Objective:**

The primary objective of this trial is to investigate the effectiveness and tolerability of methotrexate as first-line therapy in patients with pulmonary sarcoidosis compared with prednisone. The primary endpoint of this study will be the change in hospital-measured Forced Vital Capacity (FVC) between baseline and 24 weeks. Secondary objectives are to gain more insights in response to therapy in individual patients by home spirometry and patient-reported outcomes. Blood biomarkers will be examined to find predictors of response to therapy, disease progression and chronicity, and to improve our understanding of the underlying disease mechanism.

**Methods/design:**

In this prospective, randomized, non-blinded, multi-center, non-inferiority trial, we plan to randomize 138 treatment-naïve patients with pulmonary sarcoidosis who are about to start treatment. Patients will be randomized in a 1:1 ratio to receive either prednisone or methotrexate in a predefined schedule for 24 weeks, after which they will be followed up in regular care for up to 2 years. Regular hospital visits will include pulmonary function assessment, completion of patient-reported outcomes, and blood withdrawal. Additionally, patients will be asked to perform weekly home spirometry, and record symptoms and side-effects via a home monitoring application for 24 weeks.

**Discussion:**

This study will be the first randomized controlled trial comparing first-line treatment of prednisone and methotrexate and provide valuable data on efficacy, safety, quality of life and biomarkers. If this study confirms the hypothesis that methotrexate is as effective as prednisone as first-line treatment for sarcoidosis but with fewer side-effects, this will lead to improvement in care and initiate a change in practice. Furthermore, insights into the immunological mechanisms underlying sarcoidosis pathology might reveal new therapeutic targets.

**Trial registration:**

The study was registered on the 19th of March 2020 in the International Clinical Trial Registry, www.clinicaltrials.gov; ID NCT04314193.

## Background

Sarcoidosis is a multisystem, granulomatous disorder of unknown cause, which most commonly affects the lungs. Patients often have organ-specific symptoms such as dyspnea and cough, but many patients also suffer from a wide range of other disabling, non-specific symptoms including fatigue, stress, anxiety, depression, small-fiber neuropathy, decreased exercise tolerance, cognitive impairment, and chronic pain [1, 2]. These symptoms have a major impact on quality of life (QoL) and social as well as work participation of sarcoidosis patients [1, 2]. Treatment of pulmonary sarcoidosis should be considered for patients with significant pulmonary symptoms and patients with an impaired or deteriorating lung function [3, 4]. The ATS/ERS/WASOG guideline, dating from 1999, recommends oral corticosteroids as the first-choice therapy for pulmonary sarcoidosis and this is also reflected in a recent Delphi consensus statement and current practice [5–7]. These recommendations are mainly based on expert opinion and limited evidence from observational studies. Both state that more research is needed to define the best treatment for patients with pulmonary sarcoidosis [5–7]. Although corticosteroid treatment leads to short-term improvement of pulmonary function, radiological improvement, and symptom reduction, previous studies have not conclusively demonstrated a beneficial effect in preventing disease progression in the long-term [3, 4, 7]. Corticosteroids can have debilitating side-effects, such as diabetes, osteoporosis, depression and weight gain [8–10]. Long-term use of corticosteroids is associated with impaired QoL [11]. Hence, there is a major need to find better treatment options for sarcoidosis with fewer side-effects and less impact on QoL.

Methotrexate is most used as second-line therapy, when prednisone is ineffective or not tolerated, and appears to have fewer side-effects [7, 12]. Even though methotrexate is sometimes used as first-line therapy in case of (relative) contra-indications for prednisone, no data on its effectiveness as first-line treatment for pulmonary sarcoidosis exist. A number of studies evaluated the efficacy of methotrexate as second-line treatment for sarcoidosis, and showed that methotrexate had a significant steroid-sparing effect and improved pulmonary function [13–16] . Most commonly reported side-effects in these studies were gastro-intestinal complaints, headache, general malaise and infections. These side-effects only led to treatment discontinuation in a minority of cases. Moreover, according to a sarcoidosis expert survey, methotrexate seems to be safe and well tolerated when used for a prolonged period of time [12].

Tailoring of existing therapies and development of new treatments is also hampered by the incomplete understanding of the pathobiology of sarcoidosis [17]. Biomarkers predictive of disease behavior and response to therapy are highly needed to avoid overtreatment with unnecessary side-effects in some patients and undertreatment with possible organ damage as a result in others. The ideal biomarker is highly sensitive, widely reproducible, rapidly available and low priced. Unfortunately, none of the currently used biomarkers in sarcoidosis care fulfills these criteria [18]. Available data indicate a role for specific T-cells, called Th17.1-cells, and cytokine expression in dendritic cells (DCs) in predicting disease course [19]. Furthermore, higher numbers of intermediate and non-classical monocytes at baseline also seem related to response to therapy in patients treated with infliximab [20] . Further advances in serum as well as cellular biomarker discovery can provide more insights into disease mechanisms, disease behavior, and response to therapy. Home monitoring of lung function and patient reported outcomes is a more clinical approach to assess response to treatment, as this will allow for more detailed information on treatment effect [8, 21]. Together, this will contribute to personalized treatment of sarcoidosis.

This paper describes the design of the PREDMETH study, which will evaluate the efficacy of methotrexate as first-line treatment for pulmonary sarcoidosis compared to prednisone, and aims to improve evidence-based treatment options and quality of life for patients with sarcoidosis.

## Methods/design

### Objectives and endpoints

The main objective of this study is to investigate the effectiveness and tolerability of methotrexate as first-line therapy in patients with pulmonary sarcoidosis compared with prednisone. The primary endpoint of this study is the change in hospital-measured Forced Vital Capacity (FVC) between baseline and 24 weeks. Secondary objectives are to gain more insights in response to therapy in individual patients by home spirometry and patient reported outcomes. Furthermore, we aim to also examine blood biomarkers of disease progression and chronicity, to assess whether response to therapy can be predicted in individual patients, and to gain more insights into the underlying disease mechanism and potential new targets for therapy. Table 1 shows the endpoints of the PREDMETH study.

**Table 1:** Endpoints of the PREDMETH study listed as primary endpoint, secondary endpoints and explorative endpoints

Primary endpoint	
- Between-group difference in change in hospital-measured Forced Vital Capacity (FVC) % predicted between baseline and 24 weeks	
Secondary endpoints	
- Difference in change in FVC % predicted at 4, 16 weeks, 1 year and 2 years between prednisone and methotrexate group - Time to major pulmonary improvement measured by home-measured FVC, whereby major pulmonary improvement is defined as 80% of the maximum percent predicted FVC reached anywhere during the first 24 weeks of treatment - The percentage of patients with ≥5 and ≥ 10% improvement or decline in FVC at 4, 16 and 24 weeks - The percentage of patients with ≥10% improvement or decline in DLCO at 4, 16 and 24 weeks - Change over time in (Health-related) quality of life measured by King’s Sarcoidosis Questionnaire (KSQ), Global Rating of Change scale (GRoC), Chronic Respiratory Questionnaire (CRQ) and Euroqol-5D-5L questionnaire (EQ-5D-5L) at every clinical visit - Time to symptom improvement. Symptom scores measured by Visual Analogue Scales (VAS), Medical Research Council dyspnea scale (MRC) and Fatigue Assessment Scale (FAS) - Expectations with medication at baseline - Experiences and satisfaction with medication measured by the Patient Experience and Satisfaction with Medication Questionnaire (PESaM) - Number, severity and impact of side-effects compared between methotrexate and prednisone - Number of patients who discontinue/switch medication - Adherence to treatment schedule (% of patients that received at least 90% of the total cumulative dose after 24 weeks). - Correlation between angiotensin coverting enzyme (ACE), serum soluble interleukin-2 receptor (sIL-2R) and clinical parameters	
Explorative endpoints	
- Asses predictors of disease progression and response to therapy - Correlations between biomarker characteristics and clinical parameters - Change in biomarkers over time - Explore potential differences in distribution/phenotypes of monocytes, Th-cells, dendritic cells and new biomarkers identified using proteomics between patients during treatment with prednisone or methotrexate at all points	

### Design and participants

The PREDMETH study is a prospective, randomized, non-blinded, multi-center, non-inferiority trial, designed to compare effectiveness and tolerability of methotrexate versus prednisone as first-line therapy for pulmonary sarcoidosis. The study is a joint design of clinicians, scientists and a patient expert panel. In this study, 18 centers in the Netherlands will participate. The study will randomize a total of 138 treatment-naïve adult sarcoidosis patients, about to start first-line therapy for a pulmonary indication.

Patients will be informed about the study by their treating physician. If patients are willing to participate, written informed consent will be obtained, and patients will be screened for eligibility. The in- and exclusion criteria are shown in Table 2. If eligible, patients will be randomized in a 1:1 ratio to receive either prednisone or methotrexate for 24 weeks, after which they will be followed up in regular care up to 2 years. For allocation of each subject to a treatment arm a centralized electronic randomization system will be used. Hospital visits will take place at 4, 16 and 24 weeks, and for long-term follow-up after 1 year and 2 years. Additionally, patients will be asked to perform weekly home spirometry and record symptoms and side-effects via a home monitoring application for 24 weeks.

**Table 2:** Inclusion and exclusion criteria

Inclusion criteria	Exclusion criteria
- Diagnosis of sarcoidosis according to the ATS/ERS/WASOG criteria [6], in case of absent histology a diagnosis of sarcoidosis can also be established in a multidisciplinary team meeting in a sarcoidosis expert center based on a highly suggestive clinical and radiological picture [22] - Age ≥ 18 years - A pulmonary indication for treatment and parenchymal involvement on X-ray or chest CT-scan conducted within three months before inclusion (determined by the treating physician and according to current guidelines) - A forced vital capacity (FVC) of ≤90% of predicted, or a diffusion capacity of the lung for carbon monoxide (DLCO) ≤70% of predicted, or an absolute decline of ≥5% FVC decline, or an absolute ≥10% DLCO decline in the past year	- Any condition or circumstance that, in the opinion of the investigator, may make a subject unlikely or unable to complete the study or comply with study procedures - Previous immunosuppressive treatment for sarcoidosis - Use of systemic immunosuppressive therapy within the preceding three months for another disease than sarcoidosis - Pregnant, breastfeeding, or planning to become pregnant or breastfeed during the study treatment or within 90 days after the last dose in the randomized study phase. For males; planning to pro-create during the study or within 90 days after the last dose of the randomized study phase - Primary systemic treatment indication being an extra pulmonary location of sarcoidosis (e.g. cardiac of neurological) - Contra-indication for methotrexate or corticosteroids^a^

^a^Contra-indications for methotrexate or corticosteroids are defined as: severely impaired renal function (creatinine clearance < 30 ml/min), impaired hepatic function (serum bilirubin-value > 5 mg/dl or 85,5 μmol/l), bone marrow insufficiency with severe leukopenia, thrombocytopenia, or anaemia, severe acute or chronic infections, such as tuberculosis, human immunodeficiency virus, parasitic infections or other immunodeficiency syndromes, mouth, stomach or duodenal ulcers

### Hospital visits

Baseline characteristics of patients, such as gender, age, self-reported ethnicity, smoking status, comorbidity, co-medication, date of sarcoidosis diagnosis, details of organ involvement, and if available details of x-ray and chest CT scan will be recorded. At every visit, a physical examination will be performed to record weight, height, waist circumference and blood pressure of the patients. In-hospital pulmonary function measurements will include FVC, Forced Expiratory Volume in 1 second (FEV1) and diffusion capacity of the lung for carbon monoxide (DLCOc). Furthermore, routine blood samples will be collected to assess blood count, kidney- and liver function. An additional sample of 80 ml blood will be taken for analysis of Angiotensin coverting enzyme (ACE), Serum soluble interleukin-2 receptor (sIL-2R), monocyte subsets, T-cells, dendritic cells, and proteomics biomarker discovery. Patients will also be asked to complete patient-reported outcome measures (PROMs) via a home monitoring app at every visit. The PROMs consist of the Fatigue Assessment Scale (FAS), the King’s Sarcoidosis Questionnaire (KSQ), Global Rating of Change Scale (GRoC), Chronic Respiratory Questionnaire (CRQ), Patient Experience and Satisfaction with Medication Questionnaire (PESaM) and Euroqol-5D-5L questionnaire (EQ-5D-5L). At follow-up visits all adverse events will be evaluated and recorded. Side-effects will be managed according to the discretion of the treating physician. Physicians can deviate from the treatment flowchart at any time, if the clinical situation demands so. The study design is shown in Table 3.

**Table 3:**
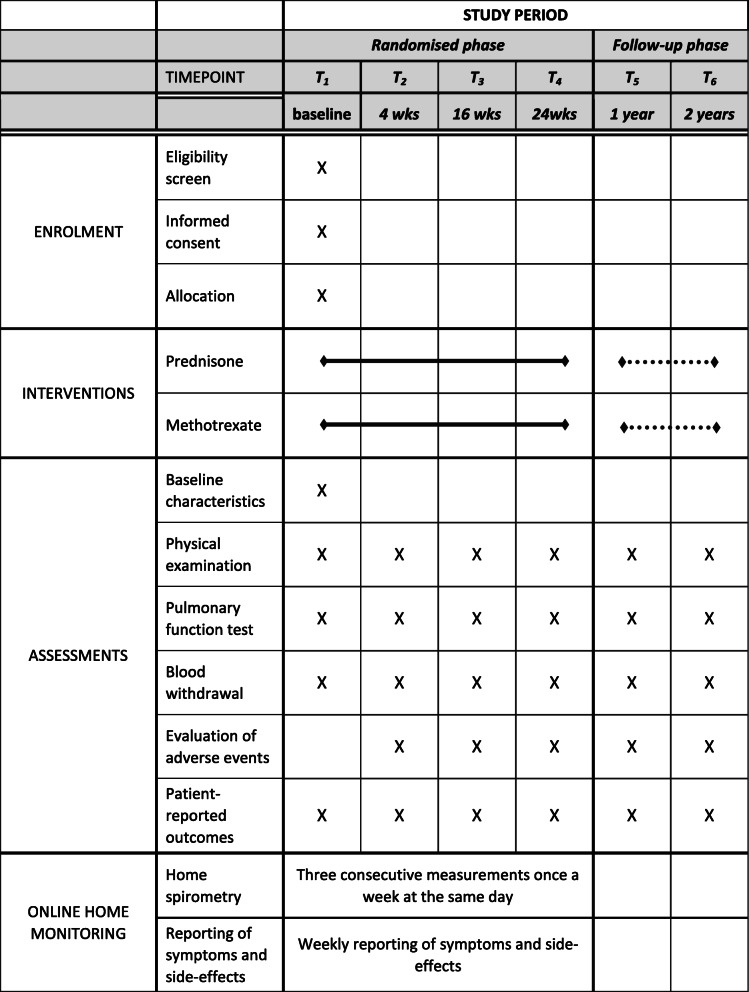
Study schedule of the PREDMETH study

### Treatment schedule

For prednisone the standard dosage according to literature is 20–40 mg daily [3, 7]. Patients will start with 40 mg prednisone daily for 4 weeks. Subsequently, this dose will be tapered to a maintenance dose of 10 mg/day within 16 weeks. The maintenance dose will be continued until 24 weeks after start of treatment. After 24 weeks, the prednisone is either continued at 10 mg or further reduced at the discretion of the treating physician. According to current clinical recommendations patients will also receive calcium 500 mg /colecalciferol 400 IE per day (6 days per week) and risedronic acid 35 mg once a week. In case of intolerable side-effects of prednisone, the treating physician will start early tapering. If side-effects remain intolerable, prednisone will be discontinued and patients will start on the methotrexate schedule.

**Fig. 1:**
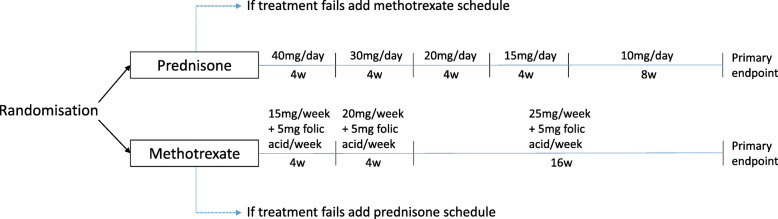
Flow chart of the treatment schedule for methotrexate and prednisone during the randomisation phase

For methotrexate, the starting dosage will be 15 mg once a week, with folic acid 5 mg once a week. Subsequently, the dose will be increased to 25 mg/week within 8 weeks if tolerated. After 24 weeks, the dose of methotrexate is either continued or reduced at the discretion of the treating physician. Methotrexate will be discontinued if aspartate aminotransferase (AST) is > 3 times the upper limit of normal. In case of intolerable side-effects of methotrexate the total dose will be divided in twice weekly. If side-effects do not subside, oral methotrexate will be switched to subcutaneous methotrexate in the same dose. If side-effects remain intolerable, dosage of subcutaneous methotrexate will be tapered according to the discretion of the treating physician. If side-effects do not subside, methotrexate will be discontinued and the patient will start on the prednisone schedule. If an absolute FVC decline of ≥10% on in-hospital measurement of FVC is detected, the other treatment arm will be added to the current therapy (i.e. patients on methotrexate will additionally start with the prednisone schedule and patients on prednisone will additionally start with the methotrexate schedule). In this case, patients will be treated with both methotrexate and prednisone and remain in the study. At any time, the treating physician may decide to deviate from this treatment schedule if the clinical situation demands so. Figure 1 shows the treatment schedule of prednisone and methotrexate.

### Home monitoring

**Fig. 2:**
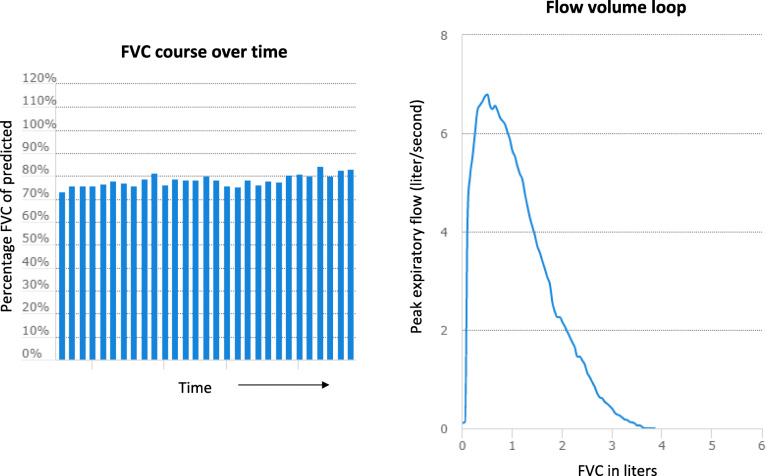
Example of Home spirometry outcome, this graphical overview is available on patient’s personal platform and is accessible for both patients and the study team

For home monitoring of pulmonary function, symptoms and side-effects, a home monitoring application will be used, which has previously been developed together with sarcoidosis patients [21]. The application will be installed on a smartphone or tablet. In this CE-marked, secured application (Curavista, the Netherlands), patients will keep track of their own data, such as pulmonary function, side-effects and symptoms. A graphic overview of results is available, so patients can gain more insight in their disease course, an example is shown in Fig. 2. A handheld spirometer (Spirobank Smart, MIR, Italy) will be handed out to all patients. Patients will be instructed to undertake spirometry (three consecutive measurements) once a week at the same day, at approximately the same time to enhance compliance and reduce variability [23]. The best result of the day will be transmitted real-time to the patients’ personal platform and will be directly accessible for both patients and the study team. This will enable close monitoring and early detection of possible deterioration. On the same day as spirometry, patients will report their symptoms and, if any, side-effects on their personal platform. Patients record cough, dyspnea, fatigue, and general complaints on a visual analog scale (VAS) and will also report dyspnea on the Medical Research Council scale (MRC). In case of side-effects, patients can record which side-effects they have and how bothersome they are. When FVC declines ≥10%, or side-effects are burdensome, an email alert will be sent directly to the treating physician, who will contact the patient within 24 h to discuss symptoms and decide whether an extra outpatient clinic visit with pulmonary function measurement is needed. Furthermore, patients will be asked to complete PROMs at every time point before the doctor’s visit.

### Serum and cellular biomarkers

Blood samples will be analyzed in order to study both serum and cellular biomarkers. With regards to serum biomarkers, the value of existing biomarkers such as ACE and sIL-2R in predicting disease progression or response to therapy will be studied prospectively. Furthermore, a proteomics approach will be used to search for possible new biomarkers in sarcoidosis. Regarding cellular biomarkers, the amount of Th17-lineage cells, DCs and monocytes will be analyzed in order to predict response for individual patients to prednisone and/or methotrexate.

### Sample size calculation

The power calculation is based on data from a previous observational study with prednisone in newly treated patients with pulmonary sarcoidosis. In this study, the mean increase in FVC with prednisone was 15%, with a standard deviation of 10.5% [8]. In a case series of 50 patients with sarcoidosis treated with methotrexate, treatment effect was in line with the findings for prednisone; 66% of patients had more than 15% improvement in vital capacity after 6 months [24]. We chose a non-inferiority margin of 5%, because FVC measurements show biological variation and thus variation < 5% is not considered clinically relevant. Based on these numbers, using a one-sided test with a power of 80% (β) and a type 1 error of 5% (α), the estimated sample size of this study is 110 patients. To allow for 20% drop-out, we aim to randomize 138 patients.

### Statistical analyses

The primary analysis will be based on the Intention-to-Treat (ITT) population, defined as all randomized patients who received at least one dose of prednisone or methotrexate. Patients who discontinue treatment prematurely will be analyzed based on the available data. As a sensitivity analysis, a Per Protocol analysis will be performed in all enrolled patients who complete the 24 week treatment regimen without major protocol violations. Change in FVC (% predicted) between baseline and 24 weeks follow-up will be compared between the two groups by using multilevel analysis with the repeated values of FVC as dependent variable and groups, time (defined as categorical variable) and baseline FVC as covariate. To account for the repeated FVC measurements, patient will be included as a random effect. Additionally the interaction terms between the time points and group will be added to assess at which point in time the groups have different average FVC values.

(Generalized) multilevel analyses will also be used to evaluate within and between-group differences in pulmonary function, symptoms, QoL scores, and side-effects over the study period. Between-group differences in mean time to onset of side-effects and time to symptom improvement will be analyzed using Kaplan Meier and log rank test. The Chi square test will be used to evaluate between-group differences in the proportions of patients who discontinue medication, proportion of patients who have FVC improvement and FVC decline, and the proportion of patients who adhere to the treatment schedule. The time to pulmonary improvement, using home spirometry, will be assessed using a linear mixed model, with FVC as dependent variable and time and treatment group as independent variables. Time will be entered as a random effect and will be modelled flexible. Moreover, this model will incorporate the change in curvature of the FVCs temporal trajectory, and thus enable estimation of the time point at which the maximum improvement in lung function is reached. Correlation between lung function, side-effects, QoL scores and symptoms will be analyzed with Pearson Correlation Coefficient. The FVC trajectory over time will be related to the trajectory of QoL scores over time with a linear mixed model. Logistic regression will be used to analyze which clinical or laboratory parameters are associated with disease progression (defined as ≥10% FVC decline) and disease chronicity.

## Discussion

There is a major unmet need for better evidence-based treatment in sarcoidosis. Although this has already been acknowledged in the ATS/ERS/WASOG guideline 20 years ago, research into first-line treatment options is still lacking. This will be the first study evaluating the efficacy of methotrexate as a first-line treatment for pulmonary sarcoidosis. As prednisone is often accompanied by many side-effects, the possibility to choose for methotrexate as first-line therapy may potentially lead to a reduction in side-effects and improvement in quality of life for patients. A non-inferiority design was chosen since we hypothesize that methotrexate is as effective as prednisone (non-inferiority margin considered), but has fewer side-effects. We chose a non-blinded design because it would be practically impossible to integrate dosing and tapering schedules as described in a double-blind study.

Previous studies evaluated the efficacy of methotrexate as second-line therapy and showed a steroid-sparing effect and significant increase in pulmonary function. In these trials, dosages between 10 and 15 mg methotrexate were used [14, 15]. In the current trial, methotrexate will be initiated at 15 mg and, if tolerated, subsequently increased until 25 mg. This treatment schedule is chosen based on experiences with methotrexate in rheumatoid arthritis, for which starting dosages of 15 mg/week escalating with 5 mg/month to 25-30 mg/week have been demonstrated to have the best clinical efficacy [25]. By using an online home monitoring program, potential side-effects will be closely monitored and where needed medication will be adjusted. The study design and treatment schedule have been developed together with a patient panel, to ensure that the burden of study participation will be acceptable for patients.

Next to side-effects and symptoms, the home monitoring program also allows for close monitoring of lung function. A previous observational study in patients with pulmonary sarcoidosis, newly treated with prednisone, showed that home spirometry can reliably detect changes in pulmonary function in sarcoidosis patients and assess the time to pulmonary improvement [8]. The major increase in FVC already occurred within the first month of treatment; however, until now no consensus exists on the optimal tapering schedule [7]. The PREDMETH trial will also provide data on time to pulmonary improvement in patients treated with methotrexate, which are currently lacking. The online home monitoring program that will be used in the current study, has been previously evaluated together with sarcoidosis patients in a pilot study. This study showed that patient satisfaction and compliance were high, and the reproducibility of FVC was good [21]. Thus, home monitoring will allow us to better evaluate (early) response to therapy, could enable personalized tapering of medication in the future and will provide more insights in the balance of effects and side-effects in newly-treated sarcoidosis patients.

Next to the important clinical aims of the study, the role of serum and cellular biomarkers in disease pathogenesis as well as predicting disease outcome will be further explored [26]. By combining these insights with the clinical outcomes of the study this trial will hopefully provide unique insights in the development and disease course of pulmonary sarcoidosis.

In conclusion, by conducting a randomized clinical trial to compare prednisone and methotrexate as first line treatment of pulmonary sarcoidosis we expect to advance evidence based treatment practice for sarcoidosis. Furthermore, seamlessly integrating translational research on patient samples from this trial will provide more insights not only with regard to tailored treatment, but also in the pathophysiological mechanisms of sarcoidosis. All together, we believe that the proposed study has the potential to enhance evidence-based and personalized therapy that improves outcomes and quality of life for patients with sarcoidosis.

## Data Availability

The full protocol and metadata will be available on reasonable request. The endpoints of the PREDMETH study are available on www.clinicaltrials.gov; ID NCT04314193.

## Abbreviations

QoL: Quality of life
DCs: Dendritic cells
FVC: Forced Vital Capacity
KSQ: King’s Sarcoidosis Questionnaire
GRoC: Global Rating of Change scale
CRQ: Chronic Respiratory Questionnaire
EQ-5D-5L: Euroqol-5D-5L questionnaire
MRC: Medical Research Council dyspnea scale
FAS: Fatigue Assessment Scale
PESaM: Patient Experience and Satisfaction with Medication Questionnaire
ACE: Angiotensin coverting enzyme
sIL-2R: Serum soluble interleukin-2 receptor
FEV1: Forced Expiratory Volume in 1 second
DLCOc: Diffusion capacity of the lung for carbon monoxide
PROMs: Patient-reported outcome measures
AST: Aspartate aminotransferase
VAS: Visual analog scale
ITT: Intention-to-Treat
